# Prophylactic effects of isomaltodextrin in a Balb/c mouse model of egg allergy

**DOI:** 10.1038/s41538-019-0057-5

**Published:** 2019-11-06

**Authors:** Yoshinori Mine, Yan Jin, Hua Zhang, Prithy Rupa, Kaustav Majumder, Takeo Sakurai, Yoshifumi Taniguchi, Ryodai Takagaki, Hikaru Watanabe, Hitoshi Mitsuzumi

**Affiliations:** 1 Department of Food Science, University of Guelph, Guelph, ON N1G2W1 Canada; 2 R&D Center, Hayashibara CO., LTD., 675-1 Fujisaki, Naka-ku, Okayama 702-8006 Japan; 3 Present Address: College of Food Engineering and Biotechnology, Tianjin University of Science and Technology, 300457 Tianjin, People’s Republic of China; 4 Present Address: Department of Food Science and Technology, University of Nebraska-Lincoln, Lincoln, NE 68588-6205 USA

**Keywords:** Immunological disorders, Cytokines

## Abstract

The aim of this study was to evaluate the potential effects of isomaltodextrin (IMD), a dietary saccharide polymer derived from enzymatically produced from starch, on the ability to alter immune response (IR) bias to hen egg ovalbumin (Ova) induced allergic inflammation in mice. Groups of Balb/c mice were pre-treated with various doses of IMD in drinking water (1.0, 2.5, and 5.0% w/v) for 6 weeks and subsequently sensitized to the Ova together with continuous administration of IMD. To evaluate changes in immune response bias, immunoglobulin isotype-associated antibody activity, concentrations of type 1 and 2 cytokines and the percentage of T-regulatory cells (T-regs) in blood were measured. Clinical signs of allergy were assessed after oral challenge with Ova. Treatment with IMD did not significantly alter the frequency of clinical signs, however there was a trend in the overall reduction of clinical signs. Effect on IR bias was observed in the treatment groups as reflected by reduction in a type 1-biased phenotype as evident by decrease in isotype-specific IgE, IgG and increase in IL-12 cytokine production and a high proportion of T-regs. This study revealed that IMD could be a useful prophylactic candidate for alteration of allergic IR bias in mice and an immune-stimulator for reducing egg induced allergic reactions.

## Introduction

Foods that may cause allergic reactions are particularly insidious. Food allergy, an adverse immunologic reaction to food, affects 6–8% of children and 4% of adults in North America.^1,2^ The prevalence of food allergy in children (aged 0 to 17 years) has slowly increased in the USA, from 3.4% in 1997–1999 to 5.1% in 2009–2011, associated the major foods of milk, eggs, fish, crustacean shellfish, wheat, soy, peanuts, and tree nuts.^3^ It has been reported that 7.5% of Canadians—7.7% of adults and 6.9% of children under 18 years of age having at least one food allergy, most commonly associated with peanut, tree nut, fish, shellfish, sesame, milk, egg, wheat, and soy.^4^ Both the prevalence and severity of food allergies have increased in past few decades.^5,6^ Egg, milk, and peanut together contribute to nearly 90% of the food allergies.^7^ A recent study by FAAN (Food Allergy and Anaphylaxis Network) and FARP (Food Allergy Research and Resource Program) reported that over 12 million Americans suffer from food allergies.^8,9^ This indicates that food allergies are occurring at an alarmingly high rate, pressing the need for efficient preventive measures. The prevalence of childhood food allergy and the duration of these allergies, particularly those considered to be transient, like egg and milk allergy continues to increase. Methods to evaluate the potential allergenicity of foods are limited and research in developing assessment tools is needed to investigate factors for allergenic susceptibility and to improve on valid animal models for food allergy. Food allergies not only affect the susceptible individuals but also have an impact on food industries especially with the labeling laws and manufacturing practices.

To reduce the risk of food allergy, anti-IgE therapy, SOTI (specific oral tolerance induction), PIT (peptide-based immunotherapy), DNA-based immunotherapy, genetically engineered egg allergens and off late probiotics have come up with promising results but needs an in-depth study.^10^ Also, currently available therapeutics (anti-histamine, epinephrine, and steroids) provide only symptomatic relief. Due to the complex nature of allergic disease, standard treatments are limited to allergen avoidance, nutritional support, and immediate access to emergency medication. Immunoglobulin E (IgE)-mediated immune response to egg proteins in food can be life-threatening, leading to responses ranging from repeated scratching, diarrhea, vomiting, and rarely resulting in death.^11^ Mainly glycoproteins from egg white have been identified as food allergens. Among all the glycoproteins, egg white Ovalbumin (Ova), and ovomucoid (Ovm) are classified as dominant allergens and play a crucial role in the egg-induced allergenic response.^12^ Ovm is heat resistant and resistant to digestive enzymes whereas Ova is easily digested and absorbed in the gut.^13^

Most individuals who suffer from food allergy are polysensitized and therefore allergic to more than one food.^14^ Allergen specific immunotherapies would not be beneficial in this case and hence the use of allergen-nonspecific therapy could circumvent this problem by enabling change in the host immune response (IR), which ideally would induce tolerance toward all food allergens. Polysaccharides have been shown to act as prebiotics with IR modulating properties for the prevention of food allergies.^15^

Prebiotics are defined as “a substrate that is selectively utilized by host microorganisms conferring a health benefit”.^16^ The criteria that are classically met by dietary prebiotics are: resistance to acidic gastric pH and mammalian digestive enzymes, as well as high absorption yield through the gastrointestinal (GI) tract. Maltodextrins are branched polysaccharides composed by a maximum of seventeen chains of dextrose molecules, linked with alpha-glycosidic bonds consisting of α (1 → 4) and α (1 → 6) linked D-glucose units. Isomaltodextrin (IMD) (GI resistant maltodextrin) is a highly branched soluble α-glucan with a relatively low degree of polymerization.^17^ This glucan partially escapes digestion in the small intestine and may undergo fermentation by bacteria in the large intestine, and therefore, it could play a role as a prebiotic.^17,18^ It is considered as generally recognized as safe (GRAS) by the US Food and Drug Administration (FDA). Fructooligosaccharides (FOSs) occur naturally in a wide variety of foods. In fact, Americans consume ~2.5 g of inulin and oligofructose daily (range of 1–4 g), mostly from wheat and onions.^19^ In terms of safety, FOSs cause few adverse effects, and the adverse effects are minor in nature.^20^

Maltodextrin and its enzymatically modified forms have been investigated for different biomedical properties. IMD was digested partially only by small intestinal mucosal membrane in healthy subjects and reduced the glucose.^21,22^ The efficacy of IMD in the treatment of intestinal inflammation was also investigated in a mouse model of colitis and resulted in a significant reduction in the expression of pro-inflammatory mediators TNF-α and IL-8, as well as pattern recognition receptor TLR4.^23^ However, the effect of IMD on allergenic response has not been elucidated yet. Therefore, the primary objective of the current project is to evaluate the effect of IMD on Ova-induced allergenic response in mice. It was hypothesized that IMD could alter the IR phenotype and expression of allergy in mice sensitized to the egg white allergen Ova.

## Results

### Clinical scores and allergenic response of mice

**Fig. 1:**
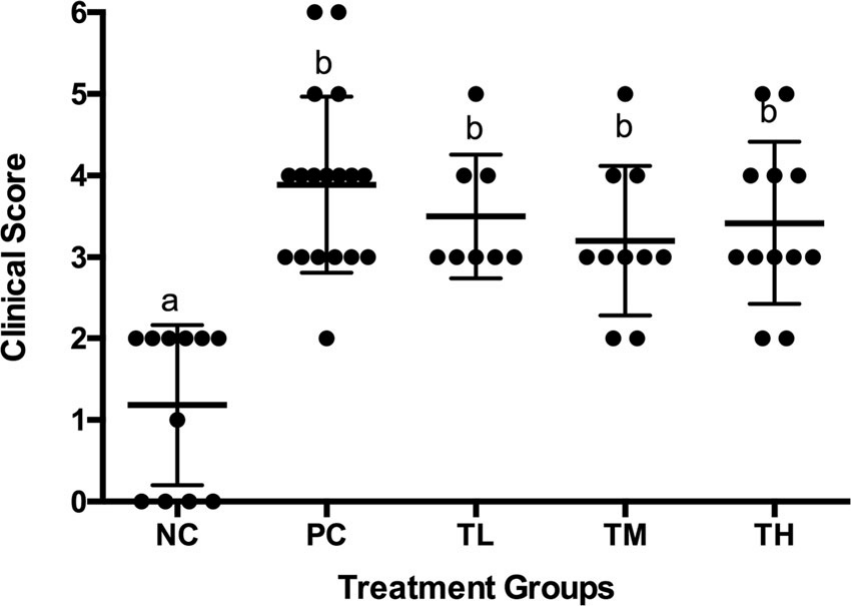
Clinical scores for individual mice. Total clinical scores for each mice post-Ova challenge were calculated. Average scores assigned by four independent observers in a blinded fashion are represented. Different letters indicate statistically significant differences

On the final experimental day, allergic reaction was induced into the mice by oral gavage by 20 mg of Ova and then the mice were observed for next 30 min for clinical scoring. Figure 1 illustrated the clinical signs observed in different groups of mice. Clinical scores were assigned and the total scores for each animal were obtained by adding scores for the individual symptoms of scratching, sneezing, isolation, diarrhea and lethargy and respiratory difficulty. Post challenge, animals in the positive control group developed statistically significant clinical scores (3.889 ± 0.254) of allergies as compared to the negative control (1.182 ± 0.296) (*p* < 0.0001). Prevention groups with IMD (Low-L, Medium-M, and High-H) (L: 3.500 ± 0.267, M: 3.200 ± 0.291, H: 3.417 ± 0.288) did not reach any statistical significance compared to the positive control group, however a trend towards a decrease in clinical scores was observed with the treatment groups compared to the positive control.

### Histamine and mast cell protease concentration

**Fig. 2:**
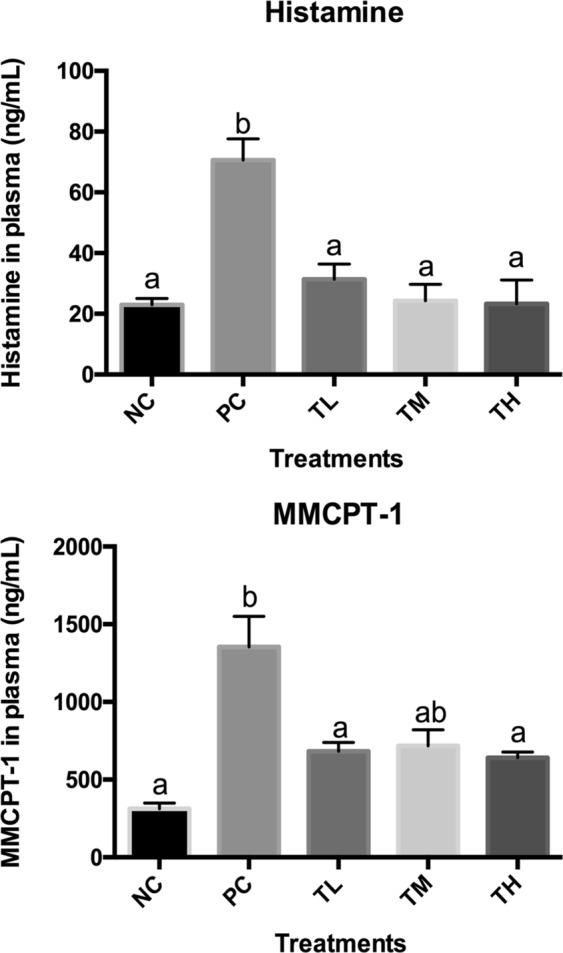
Serum histamine and mouse mast cell protease concentration. Data for serum histamine and MMCP concentration are represented as mean ± standard deviation (*n* = 6 pooled sera). Different letters indicate statistically significant differences (*p* < 0.05) between groups of mice

Histamine and mast cell protease concentration were evaluated as a measure for the mast cell activation and degranulation. Both histamine and mast cell protease concentration were increased in the positive control (Histamines: 70.598 ± 7.043 ng/mL, MMCPT-1: 1354.313 ± 196.402 ng/mL) significantly compared to the negative control group (Histamine: 22.957 ± 2.053 ng/mL, *P* < 0.0001, MMCPT-1: 312.712 ± 35.882 ng/mL, *P* < 0.001). Prevention groups of low, medium, and high-dose IMD showed statistical significant decrease in histamine (L: 31.835 ± 4.957 ng/mL, *P* < 0.001; M: 24.297 ± 5.452 ng/mL, *P* < 0.0001; H:23.251 ± 7.877 ng/mL, *P* < 0.0001) as compared to positive control group. Prevention groups with low and high-dose IMD showed statistical significant decrease in mast cell protease (L: 682.140 ± 56.801 ng/mL, *P* < 0.05; H:641.263 ± 36.807 ng/mL, *P* < 0.05) compared to positive control group. Prevention group of medium-dose IMD (717.737 ± 103.845 ng/mL) did not show statistical significant decrease in mast cell protease compared to positive control group (Fig. 2, *p* < 0.05). However, there was no significant difference observed with the IMD treated groups (*p* > 0.05) with both histamine and mast cell protease concentration as measured.

### Antibody concentrations in plasma and feces

**Fig. 3:**
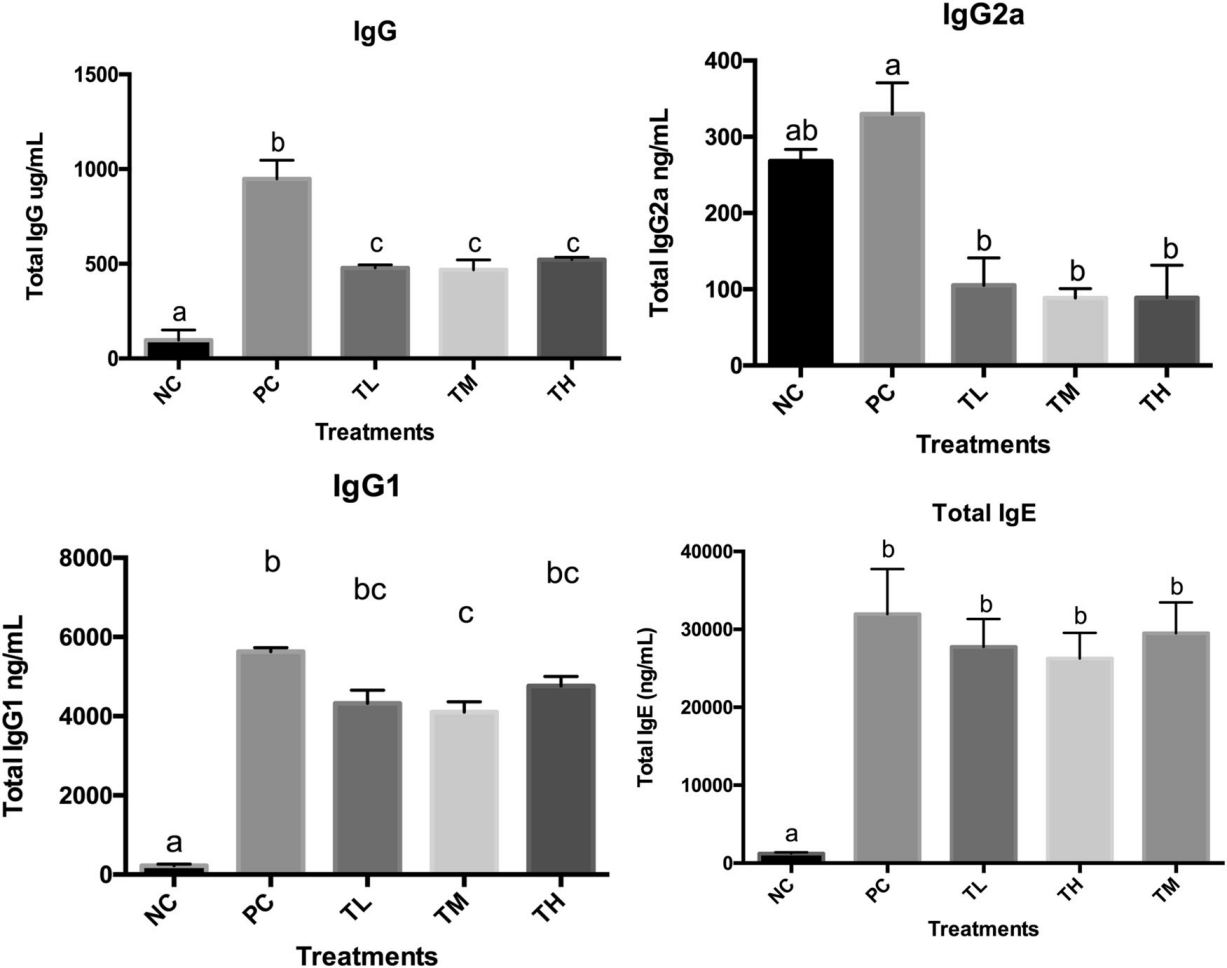
Total IgG (whole molecule), IgE, IgG1, and IgG2 related antibody activity. Rabbit anti-mouse IgG conjugated to alkaline phosphatase was used for detection of IgG and rat monoclonal anti-mouse IgE was used for IgE followed by streptavidin-HRP conjugate. Different letters indicate statistically significant differences at *p* < 0.05

**Fig. 4:**
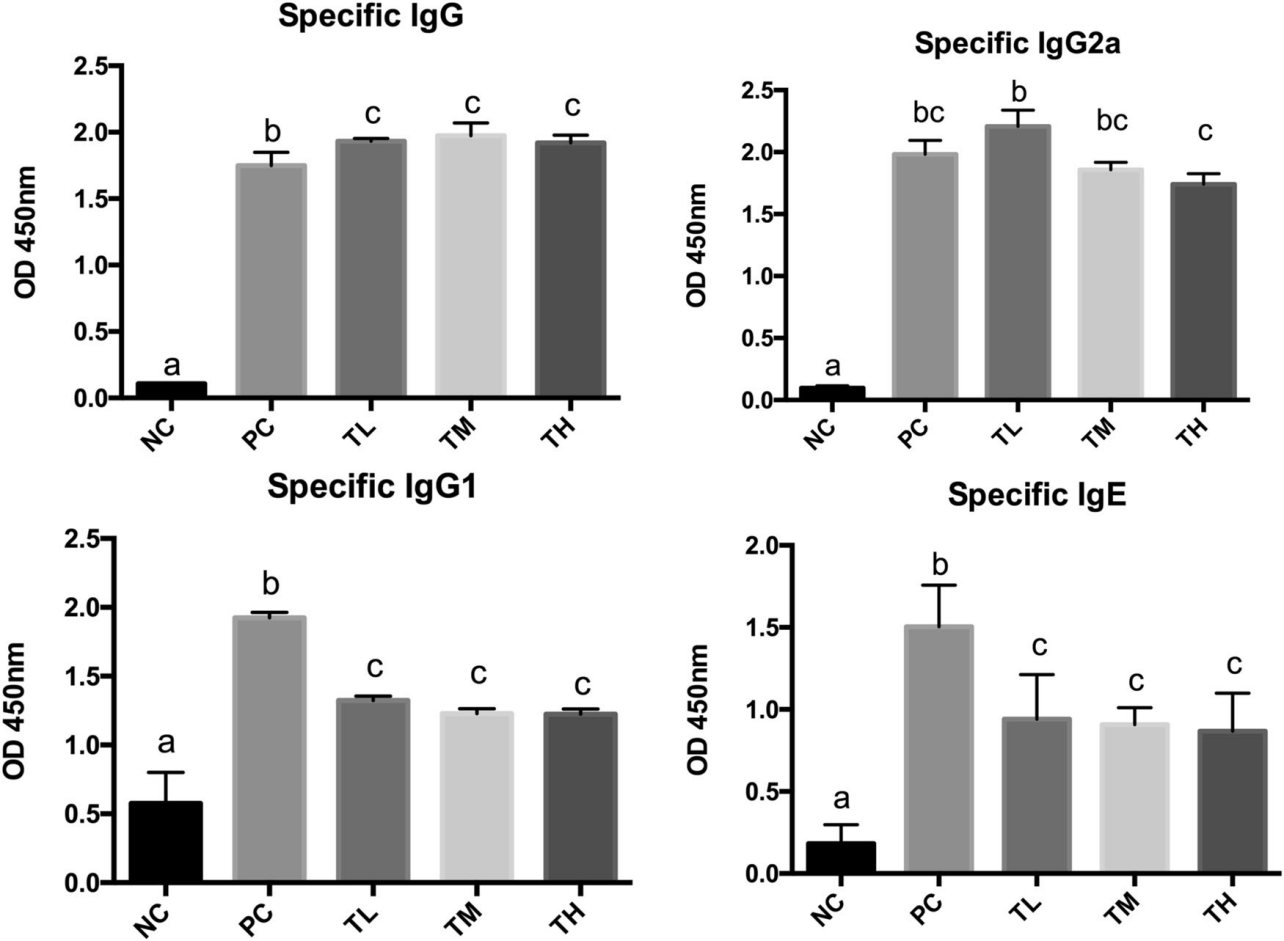
Ova-specific serum IgG, IgG1, IgG2, and IgE-related antibody activity. Rat monoclonal anti-mouse, IgG, IgG1, IgG2a, and IgE were used followed by streptavidin-HRP conjugate. Different letters indicate statistically significant differences at *p* < 0.05

**Fig. 5:**
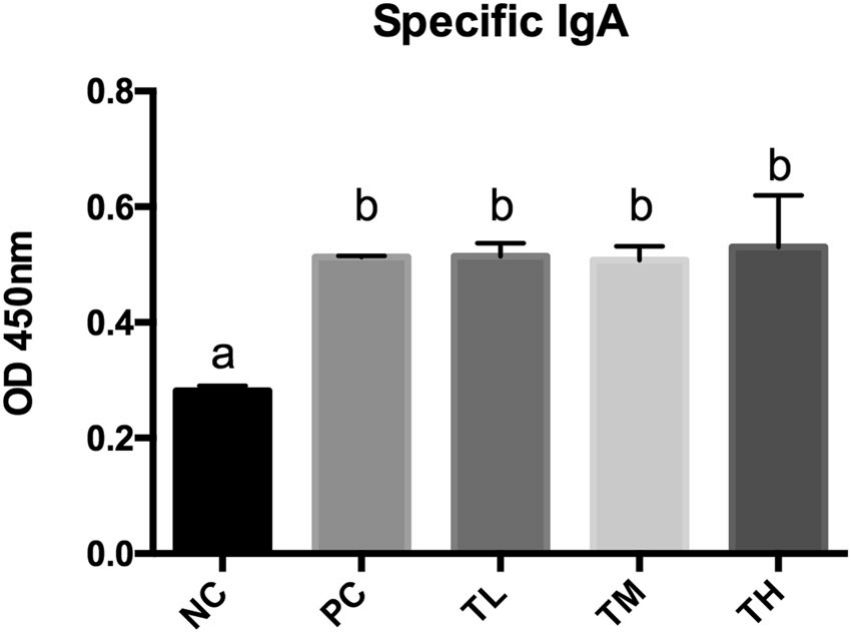
Ova-specific IgA was detected from pooled fecal samples collected from weeks 9–11 using biotinylated rat monoclonal anti-mouse IgA antibody followed by avidin-HRP conjugate. Different letters indicate statistically significant differences

Specific and total antibody levels were measured in the blood plasma. There was a significant reduction of total IgG, total IgG1, and IgG2a in all the three prevention groups (IgG L: 478.745 ± 6.409 ng/mL, *P* < 0.0001 M: 467.624 ± 21.669 ng/mL, *P* < 0.0001, H: 522.438 ± 4.696 ng/mL, *P* < 0.0001 vs. PC: 948.307 ± 28.469 ng/mL; IgG1 L: 4326.344 ± 332.243 pg/mL, *P* < 0.001 M: 4106.144 ± 255.851 pg/mL, *P* < 0.0001, H: 4766.541 ± 236.048 pg/mL, *P* < 0.05 vs. PC: 5627.638 ± 101.712 pg/mL; IgG2a L: 105.034 ± 36.056 ng/mL, *P* < 0.01, M: 88.636 ± 12.297 ng/mL, *P* < 0.01, H: 105.825 ± 48.216 ng/mL, *P* < 0.01 vs. PC: 329.994 ± 40.947 ng/mL) (Fig. 3). The total IgE showed no statistical significant difference in all three prevention groups (L: 27748.230 ± 3598.297 ng/mL, M:29495.240 ± 3975.996 ng/mL, H: 26272.100 ± 3269.893 ng/mL) compared to positive control group (31957.490 ± 5805.165 ng/mL). Specific IgG2a was significantly reduced in the high dose IMD group (1.741 ± 0.043) compared to positive control group (1.983 ± 0.033, *P* < 0.01). Specific IgG1(L: 1.324 ± 0.032, *P* < 0.0001, M: 1.230 ± 0.034, *P* < 0.0001 H:1.225 ± 0.036, *P* < 0.0001) and specific IgE (L: 0.941 ± 0.121, *P* < 0.0001 M: 0.908 ± 0.104, *P* < 0.0001, H:0.868 ± 0.095, *P* < 0.0001) levels decreased significantly after IMD pre-treatment in all three doses as compared to the positive control group (specific IgG1:1.924 ± 0.032; specific IgE: 1.504 ± 0.089) (Fig. 4). The specific IgA level was measured from the total protein extracted from the mice feces. The specific IgA showed no significant change in the prevention groups as compared to the positive control group (Fig. 5).

### Cytokine concentration

**Fig. 6:**
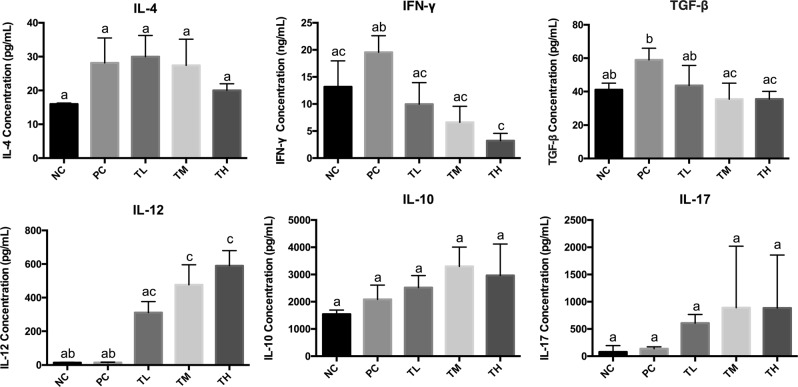
Cytokine concentration. Spleen was collected from each mouse and pooled within groups (*n* = 6) and splenocytes were isolated and cultured at 2.5 × 10^6^ cells/mL concentration of cells in triplicate wells as unstimulated (control) or stimulated with 50 μg/well of Ova for 72 h. The cytokine concentration of cell culture supernatants for IL-4, IFN-γ, IL-12, IL-10, TGF-β, and IL-17 were determined by ELISA. Different letters indicate significant difference between groups for each cytokine. Significance was taken at *p* ≤ 0.05

Cytokine expression in splenocyte was measured from the cultured spleen cells. No statistically significant difference was observed in the prevention groups as compared to the positive and negative control groups with IL-4, IL-10, and IL-17 concentrations (Fig. 6) (*p* > 0.05). Although the clear significant was not observed due to high variable of ranges, the trend of decreasing IL-4, while increasing IL-10 and IL-17 was observed. Prevention groups of medium dose (35.440 ± 3.908 pg/mL, *P* < 0.01) and high dose (35.475 ± 2.054 pg/mL, *P* < 0.05) of IMD showed statistical significantly decreased of TGF-β as compared to positive control group (58.957 ± 4.054 pg/mL). High-dose prevention group (3.226 ± 1.346 ng/mL, *P* < 0.05) significantly decreased IFN-γ compared to positive control group (19.546 ± 3.071 ng/mL). IL-12 concentration was significantly increased in a dose-dependent manner after IMD treatment with the TM (476.945 ± 119.006 pg/mL, *P* < 0.05) and TH (589.460 ± 90.797 pg/mL, *P* < 0.01) compared to PC group (13.654 ± 2.507 pg/mL) (Fig. 6).

### Percentage of Treg cells

**Fig. 7:**
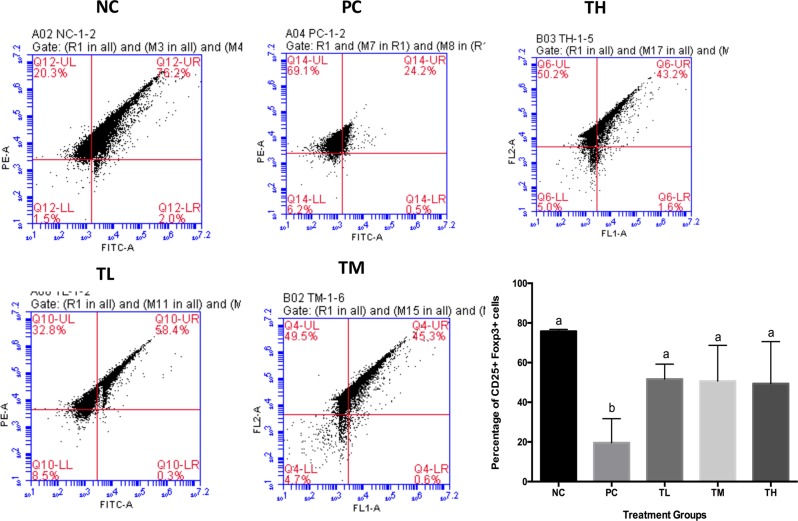
Flow cytometry. A representative image of CD25+ Foxp3+ cells for each group is shown. Percentage of CD25 + Foxp3+ cells were determined by FACS from whole blood of mice collected at the end of the experiment. Different letters indicate statistically significant differences between groups at *p* < 0.05

The flow cytometry analysis showed that the percentage of Foxp3 and CD25+ cells significantly increased in all the three prevention groups (L: 51.700 ± 3.758, M: 50.717 ± 7.349, H: 49.400 ± 9.489) (Fig. 7) as compared to those in the positive control group (19.600 ± 5.424) (*p* < 0.05).

## Discussion

Food allergy is an immunologically adverse reaction caused by foods. It is an IgE-dependent type I hypersensitivity reaction due to the imbalance of Th1/Th2. A food allergic murine model sensitized by intraperitoneally followed by the orally challenge of anti-allergic bioactive compounds was established in our past study.^24^ The present data suggested that pre-treatment with IMD (for 6 weeks) and continuous administration of IMD (week 7–11) during Ova sensitization influence the IR bias of mice undergoing allergic sensitization to Ova. The greatest effect was observed in TM and TH groups, which had an anti-allergic type 1-biased phenotype as measured by isotype-specific antibody (IgG1 and IgE) activity, relatively increased type 1-associated cytokine production (IL-12), as well as high proportion of circulating T-reg cells. Given this, it was found that mice treated with TL were more susceptible to developing clinical allergy than those treated with TM or TH. Furthermore, TH prevention group was more protective against allergy based on most of the biomarkers measured such as the decrease in specific IgG response in addition to other balance IR parameters. Thus, of all the three prevention groups, the IMD-TH treated mice displayed an increased ability to respond to Ova in terms of antibody activity (decreased specific IgG, IgG1, and IgE) and had more balanced cytokine profiles than mice otherwise treated. In order to elucidate mechanisms of anti-allergic response of IMD, we focused on Foxp3 + Treg (CD4+ CD25+ Foxp3+ T) cells. The expression of Foxp3 in mouse treated with TM and TH was increased in mice, suggesting that Treg cell polarization was promoted by IMD.

Oral immunotherapy (OIT) is a promising therapeutic approach to treat food allergic patients, however, it is generally considered as medication. Nutritional interventions may provide a new insight to improve the efficacy of OIT for food allergic patients. Dietary non-digestible oligosaccharides mimic the immunomodulatory effects exerted by human milk oligosaccharides in breast-fed infants and have been shown to reduce the risk of developing allergic diseases.^25^ It is also reported that digestible oligosaccharides can cross the intestinal epithelial barrier and directly affect immune cells involved in the process of oral tolerance induction.^26,27^ The capacity of non-digestible oligosaccharides to induce IR modulation and suppress allergic reactions in murine food allergy models suggests they may provide a potential benefit in combination with OIT strategies.^28,29^ It has been reported that oligosaccharide prebiotics have been shown to have a protective effect against allergic manifestations in high risk infants.^30^ Earlier studies have also described that IMD was digested partially only by small intestinal mucosal enzymes, and maltase and isomaltose activities were weakly inhibited.^22^ In this study the effect of IMD could only partially reduce clinical allergic signs, however there was a skewed immune response from a type 2 allergic response to a type 1 pro-inflammatory response by the TH group. These effects could be attributed to the dosage and to the digestibility of IMD by the intestinal mucosal enzymes.

Also, IMD has been confirmed to stabilize micron sized micelles in a manner similar to that of resistant maltodextrin, suggesting the inhibitory effects of IMD on the progression of micronization of micelles.^31^ Adhesion of IMD on the particle surface may have caused an increase in the surface potential.^32^ This could also be attributed to the mechanism of inhibition of allergic response in this study that may have been based on the increase on the surface potential and the dose of IMD used. Thus, it could be concluded that mice treated with TL and TM were comparatively more susceptible to developing clinical allergy than those treated with TH group which was inclined towards protecting against allergy. Hence in this study, the high dosage of IMD could be a major factor in influencing the suppression of allergic IR. The activity of IMD could also be related to the extent and mechanism of its degradation in the gut that may have influenced the suppression of the allergic response. Also, IMD is resistant to enzymatic hydrolysis, allowing it to pass into the large intestine.

Prebiotic stimulates the growth of protective commensal microbes in the gut significantly changed in infancy fecal microbiota which are associated with the development of food allergy.^33^ The proportion of abundant *Bacteroidetes, Proteobacteria*, and *Actinobacteria phyla* were significantly reduced, while the *Firmicutes phylum* was highly enriched in the food allergy group.^34,35^ A growing body of evidence suggests that gut microbiota plays an essential role in gut health and promoting local and systemic immunity. In food allergy children compare to healthy subjects, different levels of short chain fatty acids (SCFAs), in particular of butyrate, have been described.^36,37^ Thus, it is also necessary studying the effect of IMD on microbiota change, SCFAs or of other microbiota-derived metabolites production that could prevent food allergy and modulate immune system in future work. It is well known that IMD is water soluble and directly extracted from plants or made from starch and is generally regarded as safe.^27^ Hence using IMD as a prophylactic candidate for curing egg allergy is a promising approach.

In conclusion, these data provide evidence for the role of IMD in prevention of OVA allergic response in mice by inducing immune tolerance through several ways that includes a Th2-skewed to a Th1-skewed response, a regulatory response involving the transcription factor Foxp3, induction of an increase IL-12 response, and influencing mast cell functionality (suppression of histamine and MMCPT-1). This may suggest that IMD could be a potentially useful candidate for the design of a functional prebiotic food component in targeting management of food allergy.

## Methods

### Experimental design-mice and sensitization

A total of 60 mice (12 per group) were used. The mice were housed and maintained under normal husbandry conditions at the Central Animal Facility (University of Guelph). All experimental protocols were in accordance with the Canadian Council for Animal Care guidelines and all animal use protocols were approved by University of Guelph Animal Care Committee (AUP 1567).

### Pre-treatments and sensitization

**Fig. 8:**
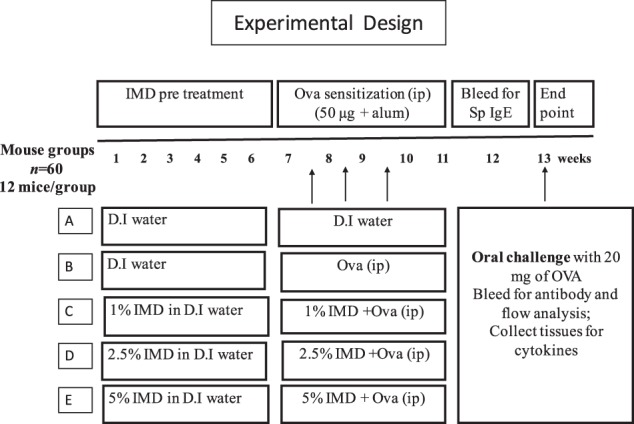
Pre-treatment and sensitization protocol. Five groups of mice, each group containing of 12 mice were used in this study. Three groups of mice were pre-treated with three different doses of IMD (1%, 2.5%, and 5%) given orally gavage for the first 6 weeks and continuously administered during sensitization (week 7–11). The negative and positive control group received just water. Mice were sensitized with 50 µg of ovalbumin (Ova) given intraperitoneally three times (i.p.). Negative control group did not receive any injection. On week 13 all mice were fasted overnight and challenged with 20 mg Ova and observed for at least 30 min for clinical signs of allergy and assigned clinical scores. Blood was taken to measure immunoglobulin isotype-associated antibody activity by enzyme-linked immunosorbent assay (ELISA) and to isolate blood mononuclear cells for culture and quantification of cytokine concentrations and to measure the proportion of circulating T-regulatory cells (T-regs) by flow cytometry

Figure 8 summarizes the pre-treatment, sensitization, sampling, and challenge schedule. Groups of mice (12 per group) were assigned to one of three prevention groups (IMD in drinking water (1.0, 2.5%, and 5.0% w/v) were administered ad lib for 6 weeks) prior to sensitization and continued during sensitization period. Group A was treated with phosphate-buffered saline (PBS; negative control). Ovalbumin (Ova: 50 μg/mice) was given by intraperitoneal injection (IP) (dissolved in 50 μL of saline and 50 μL of aluminum hydroxide gel adjuvant; Sigma-Aldrich, St Louis, MO) at weeks 7, 8, and 9. Blood was taken on week 12 to measure specific IgE as an indication of allergic sensitization and confirmed its sensitization (data not shown). Mice were allowed to fast overnight for 8 h prior to oral challenge on week 13 with 20 mg of Ova given orally via gavage. All mice were humanely euthanized after 30 min of monitoring for allergic signs. Blood was collected for various biomarker assays such as measurement of histamine, mast cell protease, antibody activity and flow cytometry analysis. Spleen was collected for measuring cytokines.

### Clinical signs

Allergic clinical signs were evaluated in a blinded fashion by four experienced independent observers and scores were assigned as described earlier.^24^ In order to be classified as allergic, mice had to exhibit clinical signs such as scratching, lethargy, gastrointestinal signs (diarrhea and bloody stool) and respiratory difficulty had to occur in combination with one of the aforementioned signs to be scored as a sign and were not themselves sufficient to identify a mouse as allergic. Thus, signs were additive, and clinical score was indicative of the severity of the allergic reaction.

### Histamine and mouse mast cell protease assay

Histamine concentrations in serum were determined by Enzyme-Linked Immunosorbent Assay (ELISA) using a commercial kit (Histamine EIA, LDN Labor Diagnostika Nord, Nordhon, Germany) as described by the manufacturer. The mouse mast cell protease enzyme (MMCP) concentration was quantified by ELISA as per manufacturer’s recommendation (eBiosciences, California, USA).

### Measurement of immunoglobulin isotype-specific antibody activity to Ova by enzyme-linked immunosorbent assay

Sera obtained on week 13 and stored at −80 °C were used to determine the Ig isotype-specific antibody activity to Ova by enzyme-linked immunosorbent assay (ELISA). Determination of individual subclasses of serum allergen-specific IgE, IgG, IgG1, IgG2a, and feces sp-IgA were examined. Optimal antigen-coating conditions were determined. Briefly, 96-well microtiter plates (Costar, Corning Inc., NY, USA) were coated with 2 µg/well of Ova (in 100 µL of 50 mM carbonate buffer; NaHCO_3_/Na_2_CO_3_, pH 9.6) for 24 h at 4 °C. Rabbit anti-mouse IgE conjugated to alkaline phosphatase was used for the detection of IgE, and rat monoclonal anti-mouse IgA was used for IgA followed by streptavidin–HRP conjugate. For IgG1 and IgG2a rat monoclonal anti-mouse IgG1 and IgG2a were used followed by streptavidin–HRP conjugate. Plates were washed 3 times with 200 μL of 0.05% Tween PBS (PBST 0.01 M, pH 7.4) per well) after coating with 2% bovine serum albumin (BSA)/phosphate buffered saline (PBS) at 37 °C for 1 h. Washing was repeated and sera diluted (1:10, 1:500 for IgE and IgG measurements, 1:100 for IgG1 and IgG2a, no dilution of IgA) in 0.05% PBST were added at 100 μL per well and incubated for overnight at room temperature with gentle shaking. For IgA, proteins were extracted using appropriate inhibitor cocktail (1 mM phenylmethanesulfonyl fluoride (PMSF), 0.1 mM ethylenediaminetetraacetic acid (EDTA), 10 µg/mL aprotinin, 10 µg/mL leupeptin and 10 µg/mL pepstatin A; Sigma-Aldrich, St Louis, MO) from feces and protein concentrations were measured according to bicinchonimic assay (BCA) assay. Plates were washed again and the bound antibodies were detected by adding respective detection antibodies at room temperature for 1 h. Specific binding activity was detected by addition of 100 µL/well of 3, 3’5, 5’-tetramethylbenzidine substrate (Sigma-Aldrich). The reaction was terminated with 1 N H_2_S0_4_ after 30 min and absorbance at 450 nm was measured by a micro titer reader (Bio-Rad 550, Hercules, USA). For total antibody similar sandwich ELISA method was used but the plates were coated by the capture antibody for each isotype.

### Cytokine analysis from mouse spleen

Individual spleens were collected at the end point post challenge aseptically from each mouse single cell suspensions were prepared. Splenocyte cell viability was assessed by trypan blue exclusion.^38^ Cells were cultured in 24 well plates in triplicate at a density of 2.5 × 10^6^ cells/mL as unstimulated (control) or stimulated with 50 μg/well of Ova. Culture supernatants were collected after 72 h incubation at 37 °C and stored in aliquots at −20 °C. Concentrations of cytokines were measured in culture supernatants using ELISA cytokine kits following manufacturer’s instructions (ebiosciences, CA, USA for IL-4, IFN-γ, IL-10, IL-12, IL-17A, and TGF-β).

### Determination of proportion of T-regulatory cells in lymphocytes of whole blood by flow cytometry

Blood collected on week 13 was also used to measure the proportion of blood T-regs from all mice by flow cytometry, based on CD25 and forkhead box P3 (Foxp3) positivity. One hundred microliters of blood from each mouse was used for double staining with anti-mouse CD25 (558642; BD biosciences, California, USA) and R-phycoerythrin (PE)-conjugated anti-mouse Foxp3 (eBiosciences, San Diego, CA), Analysis of labeled cells was performed using an Accuri Flow Cytometer (BD). Isotype and unstained controls were included to confirm specific staining of anti-CD25 and anti-Foxp3 and to adjust compensation. The antibody isotype controls used were mouse IgG1 negative control for CD25 and PE-conjugated rat IgG2a for Foxp3. Unstained samples were treated with wash buffer, PBS with 0.1% bovine serum albumin (PBA). The staining protocol was used as per the manufacturer’s instruction for whole blood sample (BD Bioscience). Live cells were gated based on forward and 90° light scatter characteristics. At least 10,000 events were acquired from each sample and all data sets were analyzed using Accuri Express software (BD Bioscience).

### Statistical analysis

Statistical calculations were performed using the GraphPad Prism 5.0 package (GraphPad Software, San Diego, CA, USA). All data were expressed as means ± SEM and subjected to ANOVA analyses followed by post hoc multiple comparison using Tukey’s test. Comparison of all the endpoint differences with a level of *P* < 0.05 was considered significant.

### Reporting summary

Further information on experimental design is available in the Nature Research Reporting Summary linked to this paper.

## Supplementary information

Reporting summary

## Data Availability

The datasets generated during and/or analyzed during the current study are available from the corresponding author on reasonable request.
